# Senescence‐induced immunophenotype, gene expression and electrophysiology changes in human amniocytes

**DOI:** 10.1111/jcmm.14495

**Published:** 2019-09-03

**Authors:** Razvan Airini, Florin Iordache, Dorin Alexandru, Lorand Savu, Florin Bogdan Epureanu, Dan Mihailescu, Bogdan Amuzescu, Horia Maniu

**Affiliations:** ^1^ Department of Biophysics & Physiology, Faculty of Biology University of Bucharest Bucharest Romania; ^2^ Department of Regenerative Medicine “N. Simionescu” Institute of Cell Biology and Pathology Bucharest Romania; ^3^ Genetic Lab S.R.L. Bucharest Romania; ^4^ Fundeni Clinical Institute Bucharest Romania

**Keywords:** amniocyte, automated patch‐clamp, flow cytometry, mesenchymal stem cell, qRT‐PCR, replicative senescence, senescence‐associated secretory phenotype

## Abstract

The aim of the study was to evidence replicative senescence‐induced changes in human amniocytes via flow cytometry, quantitative reverse‐transcription‐polymerase chain reaction (qRT‐PCR) and automated/manual patch‐clamp. Both cryopreserved and senescent amniocytes cultured in BIO‐AMF‐2 medium featured high percentages of pluripotency cell surface antigens SSEA‐1, SSEA‐4, TRA1‐60, TRA1‐81 (assessed by flow cytometry) and expression of pluripotency markers Oct4 (Pou5f1) and Nanog (by qRT‐PCR). We demonstrated in senescent vs cryopreserved amniocytes decreases in mesenchymal stem cell surface markers. Senescence‐associated β‐galactosidase stained only senescent amniocytes, and they showed no deoxyuridine incorporation. The gene expression profile revealed a secretory phenotype of senescent amniocytes (increased interleukin (IL)‐1α, IL‐6, IL‐8, transforming growth factor β, nuclear factor κB p65 expression), increases for cell cycle‐regulating genes (p16^INK4A^), cytoskeletal elements (β‐actin); HMGB1, c‐Myc, Bcl‐2 showed reduced changes and p21, MDM2 decreased. Via patch‐clamp we identified five ion current components: outward rectifier K^+^ current, an inactivatable component, big conductance Ca^2+^‐dependent K^+^ channels (BK) current fluctuations, Na^+^ current, and inward rectifier K^+^ current. Iberiotoxin 100 nmol/L blocked 71% of BK fluctuations, and lidocaine 200 μmol/L exerted use‐dependent Na^+^ current block. Transient receptor potential (TRP)M7‐like current density at −120 mV was significantly increased in senescent amniocytes. The proinflammatory profile acquired by senescent amniocytes in vitro may prevent their use in clinical therapies for immunosuppression, antiapoptotic and healing effects.

## 1 INTRODUCTION

Senescence represents an adaptive cellular phenomenon occurring in diploid cells upon repeated cell division cycles. Although to date there is no unique marker to distinguish senescent cells from differentiated non‐proliferating cells, a combination of markers characterize distinct elements of the complex chain of molecular phenomena and events leading to this phenotype.^1^, ^2^ Senescence markers include: increased expression of cell cycle regulatory genes such as *p16^INK4A^* 3, 4 or *p15^INK4B^* 5 that impede progression from G1 to S phase and trigger senescence‐associated growth arrest (SAGA); a senescence‐associated secretory phenotype (SASP) 6, 7 with increased production and secretion of proinflammatory factors such as interleukin 6 (IL‐6), IL‐8, IL‐1α, and subsequent increase in expression of a lysosomal splice variant of β‐galactosidase, senescence‐associated β‐galactosidase (SA‐β‐Gal)^8^, ^9^; the presence of senescence‐associated heterochromatin foci (SAHF)^10^, ^11^ and other epigenetic changes^12^, ^13^; activation of the DNA‐damage‐response (DDR)^14^ reflected in increased expression of p53‐binding protein 1 (53BP1) and the presence of γH2AX DNA lesion foci, initiated by phosphorylated variants of histone 2A such as γ‐H2A.X.^15^ There is a clear distinction between physiological replicative senescence (RS), involving telomere uncapping by repeated cell division, and stress‐induced premature senescence (SIPS),^1^ also called ‘stasis’,^10^ triggered by a variety of external or internal stress factors such as oncogene activation (oncogene‐induced senescence), chromatin disruption (genotoxic stress), endo/exogenous oxidative stress, X‐ray exposure or aggressive chemotherapy, endo/exogenous mitogenic signals like growth‐related oncogene α (GRO α) or circulating angiotensin II.^7^ Although short‐term senescence is beneficial by preventing cells to transform and thus propagate DNA damages, or promote transformation into tumour cells,^16^ enhancing tissue remodelling and repair, including wound healing,^17^ long‐term effects of senescent cell accumulation are detrimental, by maintenance of tissue inflammation,^18^ occupancy of stem‐cell niches,^19^ tumour promotion^20^ and a number of age‐related diseases, including atherosclerosis.^21^

Senescent cells undergo a number of metabolic changes related to molecules involved in nutrient sensing: insulin‐like growth factor 1 (IGF‐1), involved in glucose sensing, and its associated signalling pathway, mammalian (or mechanistic) target of rapamycin (mTOR), acting as sensor of high aminoacid concentrations, 5′‐adenosine monophosphate‐activated protein kinase (AMPK) and sirtuin proteins, detecting nutrient scarcity, accumulation of advanced glycation end‐products (AGE) and phospho/sphingolipids.^22^ The SASP involves activation of several signalling pathways, including mTOR, MAPK (mitogen‐activated protein kinase),^23^ phosphoinositide 3‐kinase (PI3K), and GATA4/p62‐mediated autophagy,^24^ all targeting nuclear factor κB (NF‐κB) and the CCAAT/enhancer binding protein β (C/EBPβ), which in turn transactivate transcription of numerous proinflammatory genes, such as *IL‐6*, *IL‐8*, and their receptors *IL‐6R/GP80* and *IL‐8RB/CXCR2*.^25^, ^26^, ^27^ In addition, mTOR specifically activates translation of IL‐1α, an early SASP element, which upon juxtacrine binding to its receptor activates via IL‐1 receptor associated kinase 1 (IRAK1) a positive feedback loop with further upstream NF‐κB activation.^28^ Interleukin‐6 also features an autocrine/paracrine positive feedback loop via the Janus kinase/signal transducer and activator of transcription (JAK/STAT) pathway that targets C/EBPβ, further increasing IL‐6 and IL‐8 expression.^29^ This pathway is also activated by other SASP components, like monocyte chemoattractant protein 1 (MCP‐1), vascular endothelial growth factor (VEGF), interferon I and II.^30^ Another SASP factor, transforming growth factor β (TGF‐β), in turn activates paracrine and autocrine positive feedback loops, triggering production of reactive oxygen species (ROS), DNA damage and DDR, and stable cell‐cycle arrest by induction of *p16^INK4A^* or *p15^INK4B^*.^31^ Because of deleterious effects of prolonged or excessive senescence, a number of therapies have been proposed as cures to extend lifespan and alleviate chronic age‐related diseases, focused on apoptosis induction in senescent cells, their enhanced removal by activation of immune system, SASP modulation or prevention of SAGA.^32^

Mesenchymal stem cells (MSC) of various origin (bone marrow, adipose tissue, dermis, placenta, amniotic fluid, deciduous teeth, synovial fluid or membrane, etc)^33^ represent attractive therapeutic alternatives in diseases like ischaemic stroke,^34^ brain or spine injuries,^35^ autoimmune and other diseases,^36^ graft‐vs‐host disease,^37^ because of their capacity to limit local inflammation and apoptosis and to promote neovascularisation and healing.^38^ In vitro culture and expansion are required to produce large quantities of MSC for clinical applications, but such protocols incur the risk of induction of senescence that limits or reverses their anti‐inflammatory properties.^39^, ^40^, ^41^ Several studies explored different molecular changes associated with in vitro or in vivo senescence of MSC, including proteasome activation and autophagy,^42^, ^43^, ^44^ effects of oxidative stress,^40^, ^41^ DDR and SAGA 45, 46 in either control conditions or by induction of senescence via oxidative stress, doxorubicin, X‐ray exposure or replicative exhaustion.^42^

In a previous study 47 we demonstrated multiple differences between cryopreserved and senescent amniocytes including senescence‐induced reduction in cell surface markers CD44, CD90, CD105, CD133 with preserved expression of CD29 and HLA‐ABC, highly increased expression in senescent cells of inflammation‐related genes (interferon γ) and telomerase reverse transcriptase, changes in various ion currents surface densities and proportions of cells expressing them. Despite the relatively abundant literature describing protocols to induce senescence in various stem cell preparations and senescence‐associated signalling pathways and their possible pharmacological modulation, there is an almost complete ignorance regarding the correlations between senescence‐induced signalling and metabolic changes and the electrophysiology properties of senescent stem cells and roles played by different ion currents during senescence. Therefore, the aim of the present study is to further explore activation of specific signalling pathways associated with SASP and SAGA in our in vitro senescence model, and to correlate them with changes in ion currents assessed via automated or manual patch‐clamp.

## 2 MATERIALS AND METHODS

### 2.1 Amniotic fluid cell culture

For amniocytes isolation and culture we used methods similar to those previously described.^47^ Briefly, amniocytes were obtained from human amniocentesis samples at Genetic Laboratory S.R.L, upon prior written informed consent, in accordance with EU regulations and the principles of the Declaration of Helsinki, and upon approval of ethics committee of Genetic Laboratory S.R.L, and cultured in BIO‐AMF‐2 medium (Biological Industries, Cromwell, CT), in six‐well plates, in a humidified CO_2_ incubator, with medium changes thrice weekly. Cryopreserved amniocytes were obtained after up to three passages. Cells were placed in 10% dimethylsulfoxide (DMSO), stored in liquid nitrogen, and used during first week after thawing and replating. Senescent amniocytes were kept for six consecutive weeks in culture without passages. The experiments presented in this study were performed on cryopreserved amniocyte cultures from amniotic fluid samples of n = 11 patients and senescent amniocyte cultures from amniotic fluid samples of n = 8 patients.

### 2.2 Cell proliferation assay by EdU incorporation

We assessed cell proliferation by measuring the amount of incorporation of 5‐ethynyl‐2′‐deoxyuridine (EdU) during DNA synthesis via a fast ‘click chemistry’ coupling of a fluorescent dye (AlexaFluor^®^ 488) using a standard Click‐iT^®^ EdU Kit for flow cytometry (C35002; Molecular Probes, ThermoFisher Scientific, USA) according to manufacturer's instructions. Briefly, cryopreserved and senescent amniocytes plated in subconfluent conditions in 60‐mm Petri dishes were incubated 24 hours with EdU 10 μmol/L, rinsed in 1% bovine serum albumin (BSA) in PBS, fixed 15 minutes in 4% paraformaldehyde in PBS, washed with 1% BSA in PBS, permeabilized 15 minutes with saponin‐based permeabilization and wash solution, incubated for 30 minutes in Click‐iT^®^ reaction cocktail, centrifuged and resuspended in 500 μL saponin‐based solution for flow cytometry, which was performed with a Beckman‐Coulter Gallios equipment.

### 2.3 Senescence‐associated β‐galactosidase staining

We used the SA‐β‐Gal staining method described by Dimri et al.^8^ Amniocytes cultured in 60‐mm diameter Petri dishes were washed gently twice with standard PBS, fixed in 2% formaldehyde plus 0.2% glutaraldehyde in PBS at pH 7.4 for 6 minutes, washed three times in PBS, then exposed for 12‐16 hours at 37°C to X‐Gal working solution, containing 1 mg X‐Gal substrate per ml (5‐bromo‐4‐chloro‐3‐indolyl‐β‐D‐galactoside from 20 mg/mL stock solution, BG‐3‐G; Sigma‐Aldrich), a redox system composed of potassium ferro‐ and ferricyanide (5 mmol/L each), 40 mmol/L pH 6.0 citrate buffer (17.9 mL citric acid 0.1 mol/L plus 32.1 mL dibasic sodium phosphate 0.2 mol/L per 100 mL), NaCl 150 mmol/L and MgCl_2_ 2 mmol/L. Stained cells were placed in 70% glycerol in pure water, examined and photographed with a standard inverted microscope (Olympus CKX41), and stored at 4°C in a refrigerator.

### 2.4 Surface markers assessment by flow cytometry

The expression of cell surface markers was assessed by flow cytometry (Gallios, Beckman‐Coulter) using 1 × 10^5^ amniocytes stained with fluorochrome‐conjugated (FITC—fluorescein‐isothiocyanate and PE—phycoerythrin) primary antibodies against CD29, CD31, CD45, CD44, CD49e, CD54, CD56, CD73, CD90, CD105, CD117, CD133, CD146 (Beckman‐Coulter) and SSEA‐1, SSEA‐4, TRA1‐60, TRA1‐81 (ThermoFisher Scientific). Amniocytes were detached using accutase (Sigma‐Aldrich, USA) and washed in PBS solution. Cells were then incubated with the primary antibodies at room temperature in the dark for 30 minutes. Furthermore, the cells were washed twice and centrifuged at 400*g*, 10 minutes, in PBS with 1% BSA. For negative controls, amniocytes were stained with the corresponding isotype‐matched IgG antibodies (Beckman‐Coulter). Flow cytometry data were analysed using the Gallios software (Beckman‐Coulter).

### 2.5 Expression studies via qRT‐PCR

Gene expression levels in cryopreserved and senescent amniocytes were assessed by qRT‐PCR. Total cellular RNA was isolated from cultured cells using RNeasy Mini Kit (Qiagen, Hilden, Germany) and reverse‐transcription reaction was performed with M‐MLV polymerase High‐Capacity cDNA Reverse Transcription kit (ThermoFischer Scientific, USA). mRNA levels of senescence‐associated genes (*IL‐1α*, *IL‐6*, *IL‐8*, *TGF‐β*, *NF‐κB p65*, *eNOS*, *HMGB1*, *p16^INK4A^*, *β‐actin*), pluripotent stem cell markers (*Oct4*, *Nanog*), and housekeeping gene *GAPDH* were quantified using SYBR Green method (SYBR Select Master Mix; ThermoFischer Scientific). The senescence pathways in amniocytes were investigated by quantifying mRNA levels of *p21*, *MDM2*, *c‐Myc*, *Bcl‐2* using TaqMan hydrolysis probes (Applied Biosystems). qPCR reactions were carried out in a real‐time thermocycler (ViiA7; Applied Biosystems, USA), following manufacturer's guidelines. The results were expressed using relative quantitation ($2^{-\Delta C_{\text{T}}}$), where ∆*C*_T_ represents *C*_T_ difference between values for senescent and cryopreserved amniocytes. Primers used for qRT‐PCR assays are listed in Table S1.

### 2.6 Electrophysiology and pharmacology assays via automated/manual patch‐clamp

Automated patch‐clamp electrophysiology and pharmacology experiments were performed at room temperature with a CytoPatch™2 instrument driven by the CytoLogic control software (Cytocentrics Bioscience GmbH, Germany). This system applies the Cytocentering technology.^48^ For experiments we used standard two‐channel microfluidic chips with quartz micropipette tips having 2.5‐µm inner diameter. Cells were detached with accutase, centrifuged and resuspended in extracellular solution. For each experiment 3 µL of cell suspension were placed in the transport channel of the chip by the dispensing needle of the instrument. For manual patch‐clamp experiments we used borosilicate glass capillaries (GC150F‐10; Harvard Apparatus, USA) pulled with a PUL‐100 equipment (WPI, Sarasota, FL) and fire‐polished with a home‐made microforge to yield a resistance in solution between 2‐3 MΩ. The patch‐clamp setup included an inverted microscope placed on an antivibratory platform in a Faraday cage, a stage temperature controller (TC202A; Harvard Apparatus, MA, USA), a resistive feedback amplifier (WPC‐100; ESF electronic, Göttingen, Germany) connected to a Digidata 1322A AD/DA interface controlled by the pClamp8.2 software (Axon Instruments, Molecular Devices, Sunnyvalle, CA). The electrophysiology assay included several voltage‐clamp protocols identical to those applied in our previous study by manual patch‐clamp^47^ for comparison. A general protocol for K^+^ currents consisted in 300‐ms voltage steps from −60 to +60 mV in 10‐mV increments from a holding potential of −80 mV. A protocol for fast Na^+^ currents consisted in 6‐ms steps from −60 to +40 mV in 10‐mV increments from a holding potential of −100 mV. A double‐ramp voltage protocol consisted in 2‐second ramps from −120 to +80 mV and back to −120 mV, from a holding potential of −80 mV. The same protocol was used to study TRPM7‐like currents. We defined a special voltage protocol for use‐dependent block of Na^+^ current by lidocaine, consisting in 40 6‐ms depolarizing steps at −20 mV, with a frequency of 30 Hz, from a holding potential of −140 mV.

### 2.7 Solutions and chemicals

For identifying different ion current components in cryopreserved and senescent amniocytes we used the same solutions as in our previous study.^47^ The extracellular solution contained (in mmol/L): NaCl 135, KCl 5.4, CaCl_2_ 1.8, MgCl_2_ 0.9, NaH_2_PO_4_ 0.33, HEPES 10, D‐glucose 10, pH 7.40 at 25°C with NaOH. The internal (pipette) solution was composed of (in mmol/L): KCl 140, EGTA 5, HEPES 10, pH 7.21 at 25°C with KOH. For the study of TRPM7‐like currents we prepared solutions similar to those used in our previous studies^49^: external solution (in mmol/L): NaCl 135, CsCl 5.4, CaCl_2_ 1.8, MgCl_2_ 0.9, NaH_2_PO_4_ 0.33, HEPES 10, D‐glucose 10, pH 7.40 at 25°C with NaOH; internal solution (in mmol/L): Cs gluconate 130, CsCl 25, MgCl_2_ 1, Na_2_ATP 5, Na_2_GTP 0.1, EGTA 1, HEPES 5, pH 7.21 at 25°C with CsOH. The osmolality of external solutions was checked and adjusted to 330 mOsm/kg H_2_O with sucrose 1 mol/L. A divalent cation‐free (DVF) external solution for TRPM7 experiments was prepared by omitting to add salts of these divalent cations. All solutions were filtered with 0.22 µm filters, and external solution was degassed prior to use. We applied the following pharmacological compounds: iberiotoxin 0.1 µmol/L (STI‐400; Alomone Labs, Jerusalem, IL), lidocaine 200 µmol/L (L‐145; Alomone Labs), and nifedipine 1 µmol/L. Iberiotoxin working solution was prepared from a 100 µmol/L stock solution in external solution, lidocaine hydrochloride from a 100 mmol/L stock solution in pure water, and nifedipine from a 10 mmol/L stock solution in pure ethanol. All chemicals, except otherwise specified, were from Sigma‐Aldrich or Merck.

### 2.8 Data analysis

All experiments described in this study were performed on samples (amniocyte cultures) from a single patient. Pooled samples were not used. Data are reported as mean ± SD or mean ± SEM, n indicating the number of samples. Statistical significance was assessed using either two‐tailed Student's *t* test for independent samples or its non‐parametric variant (Mann‐Whitney test), according to results of normality tests, for quantitative data, and two‐tailed Fisher's exact probability test for categorical frequency data. For all statistical tests we set a critical level *α* = 0.05. The datasets generated during the current study are available from the corresponding author on reasonable request.

## 3 RESULTS

### 3.1 Proof of RS of amniocyte cultures via lack of EdU incorporation and SA‐β‐Gal staining

**Figure 1:**
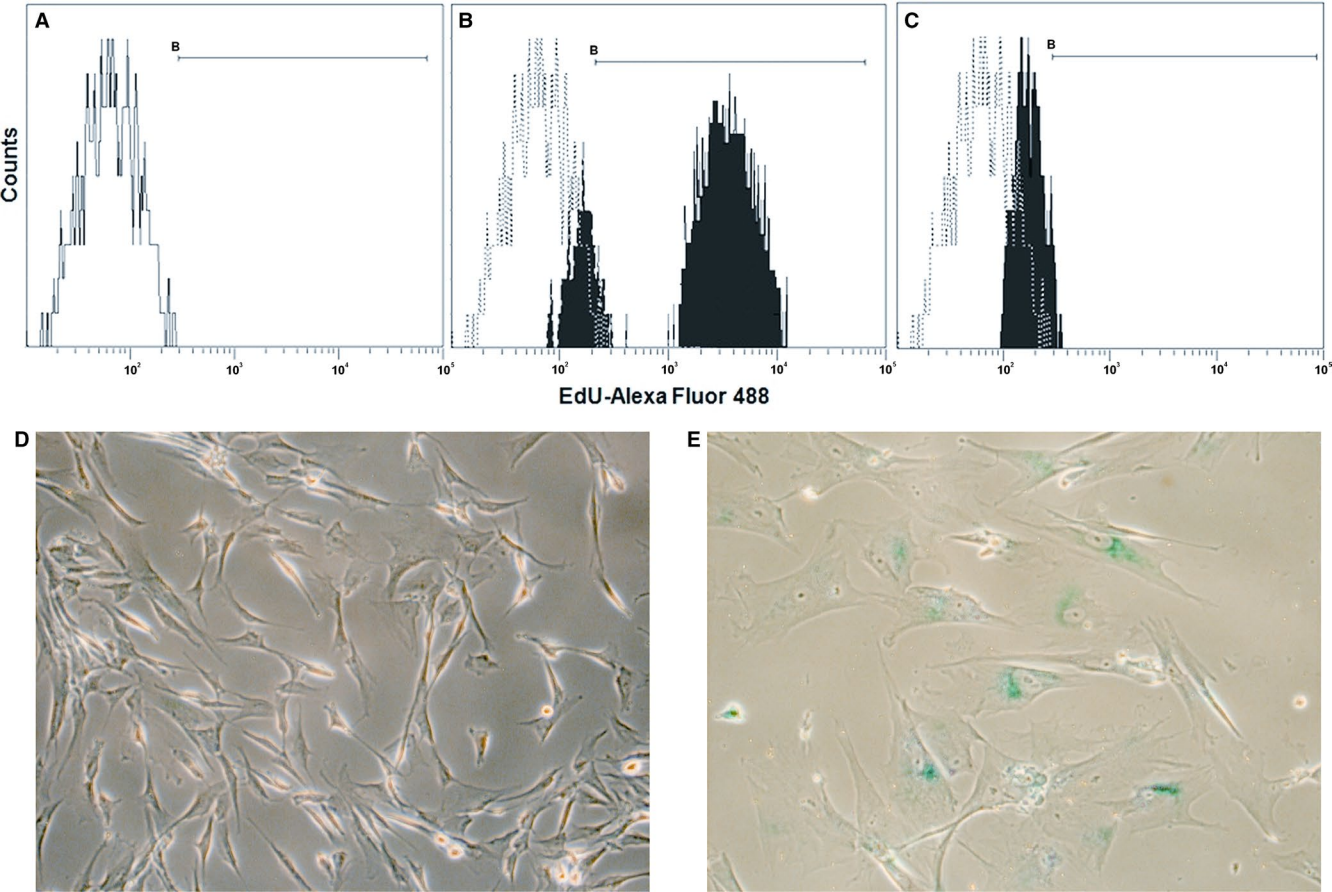
Proof of replicative senescence of amniocyte cultures. (A‐C) Staining of amniocytes treated with 5‐ethynyl‐2′‐deoxyuridine (EdU) 10 μmol/L for 24 h via Click‐iT^®^ reaction with AlexaFluor^®^ 488 and flow cytometry: (A) negative control of unstained cryopreserved amniocytes; (B) cryopreserved amniocytes show 58% EdU incorporation during S‐phase DNA synthesis; (C) senescent amniocytes (replated at low density) lack EdU incorporation; (D, E) staining for SA‐β‐Gal of cryopreserved (D) and senescent (E) amniocytes (×40 objective, phase contrast microscopy)

We tested for the presence of cell proliferation in amniocyte cultures via EdU incorporation (10 μmol/L for 24 hours), followed by Click‐iT^®^ reaction with AlexaFluor^®^ 488 and flow cytometry (Figure 1A‐C). Cell cultures loaded with EdU were tested in two technical replicates for each condition. While cryopreserved amniocytes featured high percentages of EdU incorporation during S phase of cell cycle, this phenomenon was absent for senescent amniocytes. We also proved senescence via the widely used SA‐β‐Gal staining method applied to amniocytes cultured on 60‐mm Petri dishes (Figure 1D,E). After 24 hours of incubation at 37°C with the X‐Gal substrate‐containing staining solution, senescent amniocytes, which have been replated at low density, showed specific blue staining, while this phenomenon was absent for cryopreserved amniocytes. The percentages of cells featuring characteristic SA‐β‐Gal staining (obtained by counting >200 cells for each condition) were 7% for cryopreserved vs 93% for senescent amniocytes.

### 3.2 Cell surface markers assessed by flow cytometry

**Figure 2:**
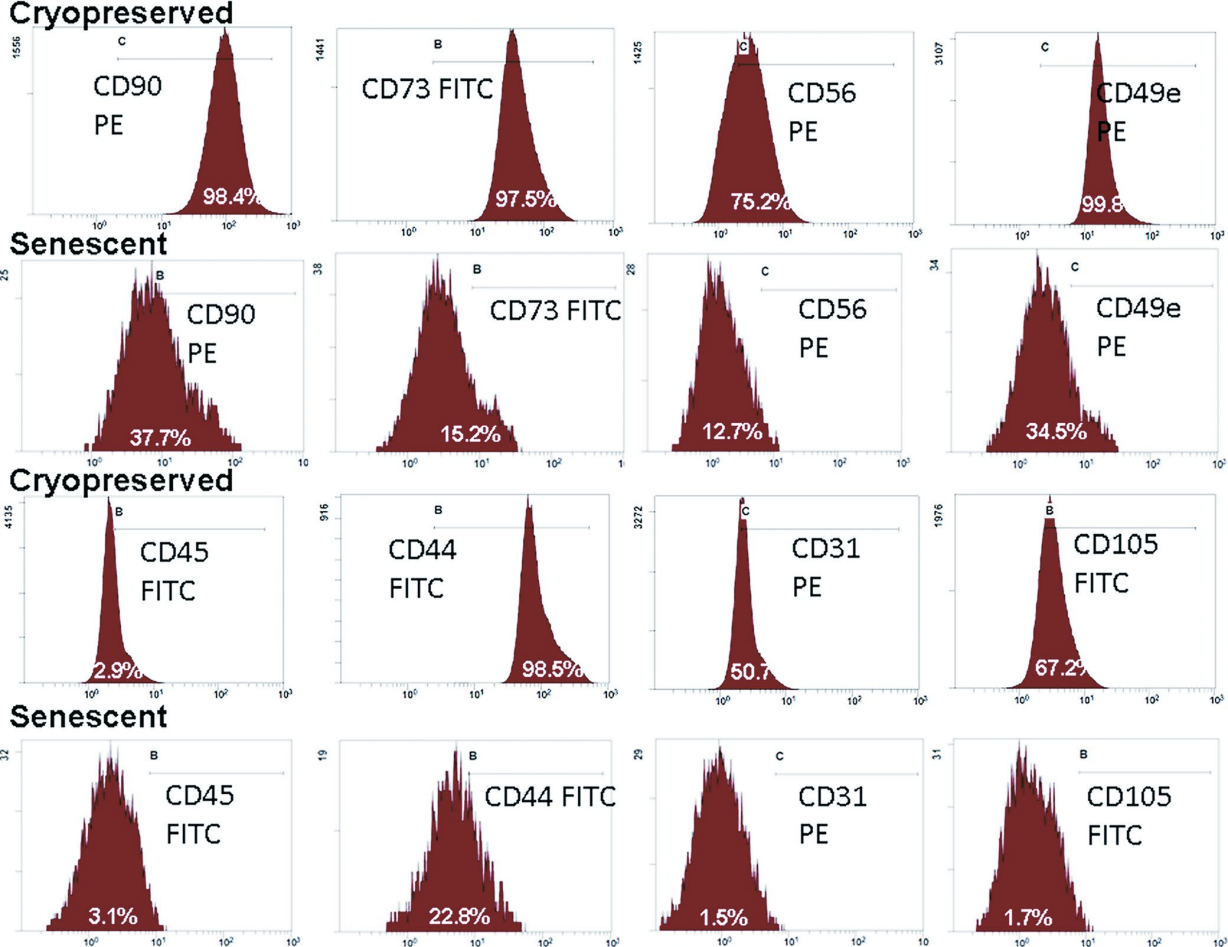
Flow cytometry assays for stem cell markers CD31, CD44, CD45, CD49e, CD56, CD73, CD90 and CD105 in cryopreserved and senescent amniocytes. Percentages of positive cells for each marker are indicated on the corresponding distribution histograms. See also Table S3 and Figures S1 and S2 of Supporting Information

Characterization of cryopreserved and senescent amniocytes in order to evidence changes in immunophenotype was performed by flow cytometry. Our results show that cryopreserved amniocytes express the mesenchymal markers CD29 (98.4%), CD31 (50.7%), CD44 (98.5%), CD49e (99.8%), CD54 (10.3%), CD56 (75.2%), CD73 (97.5%), CD90 (98.4%), CD105 (67.2%), CD146 (88.7%), and do not express CD45 (2.9%), CD117 (4.9%) or CD133 (1.7%) (Figure 2; Figure S1 and Table S3 of Supporting Information). The absence of CD45, CD117 and CD133 expression shows that there were no haematopoietic stem/progenitor cells at the time of amniocentesis (16‐20 weeks), when these cells are no longer present in amniotic fluid.^50^ The expression of surface markers decreased in senescent amniocytes by up to 82.3% compared to cryopreserved amniocytes, as follows: CD29 54.8%, CD31 1.5%, CD44 22.8%, CD49e 34.5%, CD54 8.5%, CD56 12.7%, CD73 15.2%, CD90 37.7%, CD105 1.7%, CD146 58.3% (see Table S3 of Supporting Information). Pluripotency cell surface markers were positive for high percentages of cells in both preparations, as follows: for cryopreserved amniocytes SSEA‐1 73.94%, SSEA‐4 84.05%, TRA1‐60 92.9%, TRA1‐81 90%, and for senescent amniocytes SSEA‐1 58.3%, SSEA‐4 71.5%, TRA1‐60 91.35%, TRA1‐81 84.55% (Table S3 and Figure S2 of Supporting Information).

### 3.3 Gene expression studies by RT‐PCR

**Figure 3:**
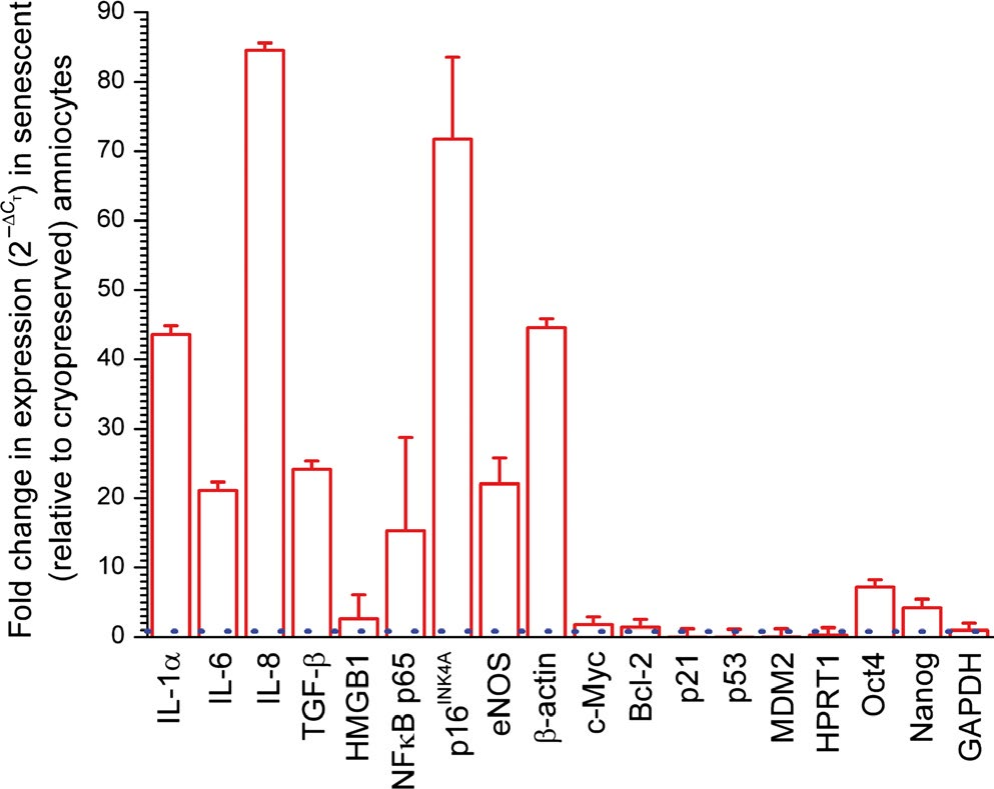
Differences in gene expression levels between senescent and cryopreserved amniocytes. The fold‐change in expression in senescent amniocytes (taking expression levels in cryopreserved amniocytes as reference) was computed as $2^{-\Delta C_{\text{T}}}$. Error bars represent SD of multiple replicates in senescent amniocytes computed vs average values in cryopreserved amniocytes. The equal expression level is marked with a horizontal dashed line. See also Tables S1 and S2 of Supporting Information. IL, interleukin; NF‐κB, nuclear factor κB; TGF‐β, transforming growth factor β

After six consecutive weeks in culture without passage, senescent amniocytes featured increased expression of *IL‐1α*, *IL‐6*, *IL‐8*, *TGF‐β*, *NF‐κB* (*p65*). Cell cycle‐regulating genes such as *p16^INK4A^*, *eNOS* and cytoskeletal elements such as *β‐actin* also showed increased expression, while *HMGB1*, *c‐Myc*, *Bcl‐2* showed little change, and *p21*, *MDM2* and the housekeeping gene *HPRT1* featured decreased expression (Figure 3; Table S2 of Supporting Information). Pluripotent stem cell markers *Oct4* (*Pou5f1*) and *Nanog* were expressed in both cryopreserved and senescent amniocytes, while the housekeeping gene *GAPDH* featured almost equal levels of expression in both preparations (Figure 3; Table S2 of Supporting Information).

### 3.4 Identification of multiple ion currents by patch‐clamp and pharmacology assays

**Figure 4:**
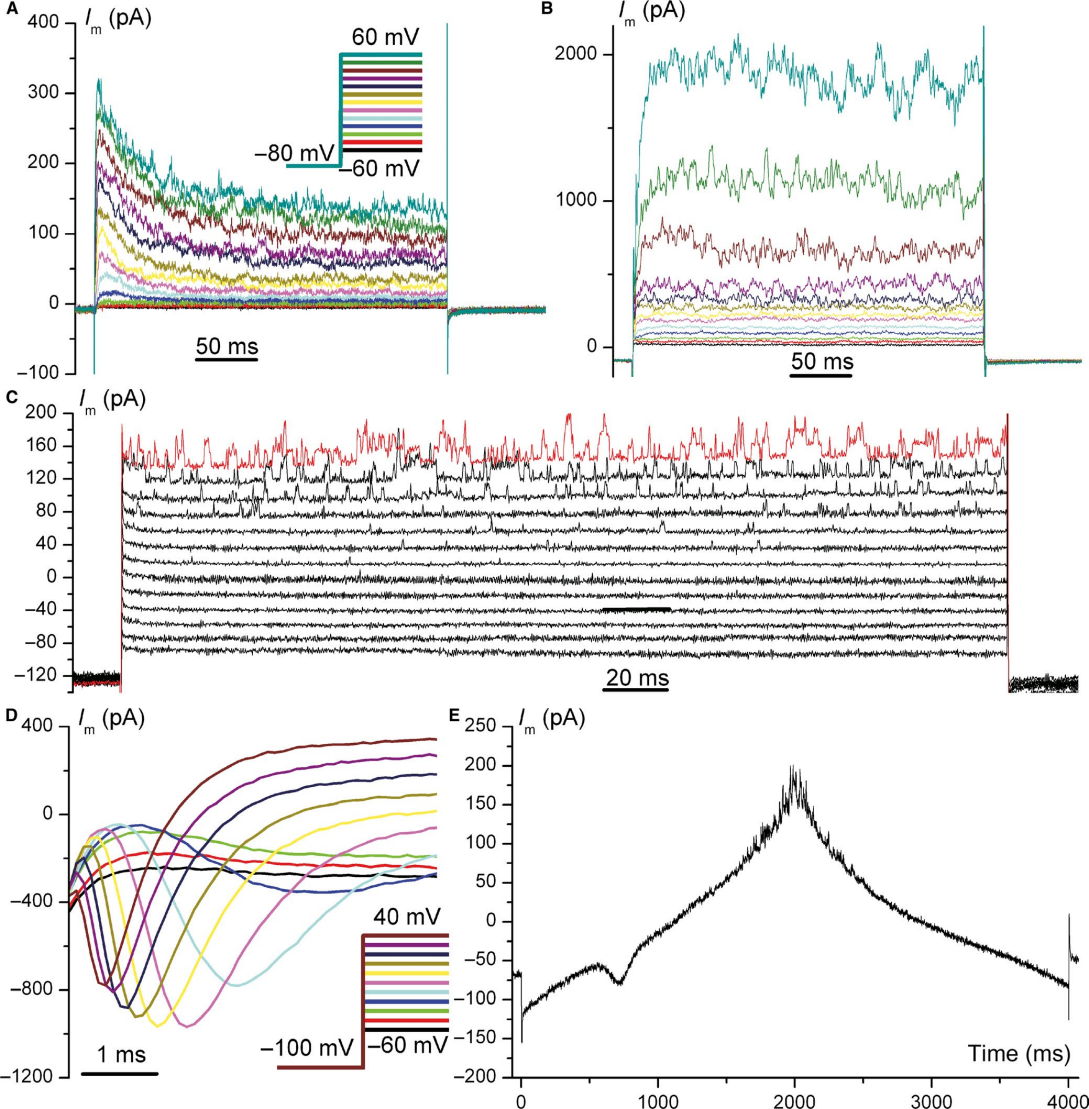
Ion currents recorded in amniocytes. (A and B) Two voltage‐clamp recordings in senescent amniocytes via automated patch‐clamp, using a general protocol for K^+^ currents (voltage steps shown in insert of A). *I*_or_ and big conductance Ca^2+^‐dependent K^+^ (BK) fluctuations are visible in both recordings, while *I*_A_ can be noticed only in (A); (C) same protocol applied to a cryopreserved amniocyte with very small current levels; single‐channel BK openings can be noticed at larger depolarizations (+20 to +60 mV); (D) voltage‐dependent Na^+^ current (*I*_Na_) in a senescent amniocyte (voltage protocol shown in insert); (E) double‐ramp voltage‐clamp protocol (from −120 to +80 mV and back) applied to a senescent amniocyte; a small T‐type Ca^2+^ channel‐like current with a threshold of ~−50 mV is present on the ascending ramp*. I*_A_, inactivatable A‐type K^+^ current; *I*_or_, outward rectifier K^+^ current

**Figure 5:**
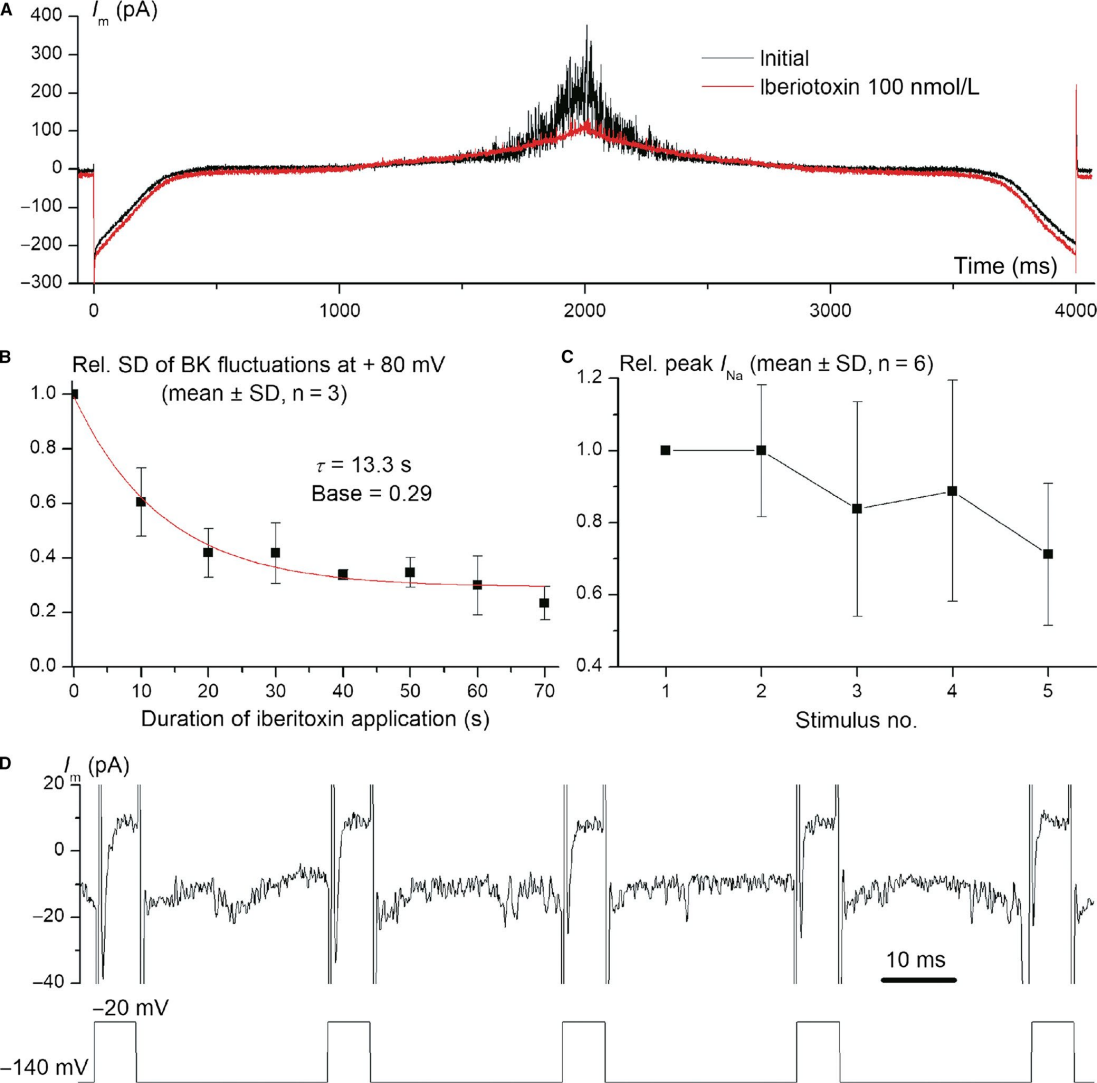
Pharmacology assays in cryopreserved amniocytes via automated patch‐clamp. (A) Double‐ramp voltage‐clamp protocol with traces recorded before and during application of iberiotoxin 100 nmol/L that blocked big conductance Ca^2+^‐dependent K^+^ (BK) current fluctuations at positive potentials; (B) kinetics of BK channels block by iberiotoxin: SD of current fluctuations in a 6‐ms interval centred around the +80 mV peak in the double‐ramp protocol (values relative to the sweep before compound application, mean ± SD of n = 6 experiments). Fitting with a monoexponential function yielded a time constant *τ* = 13.3 s and a base (constant) of 0.29; (C) kinetics of use‐dependent *I*_Na_ block by lidocaine 200 µmol/L (relative peak *I*_Na_ amplitudes for five consecutive depolarizing pulses, mean ± SD of n = 3 experiments); (D) voltage‐clamp protocol for study of use‐dependent *I*_Na_ block by lidocaine (only the first five of 40 consecutive pulses are shown). Applied voltage is shown below the current trace. *I*_Na_, voltage‐dependent Na^+^ current

We performed automated whole‐cell patch‐clamp experiments on n = 17 cryopreserved amniocytes, using three voltage‐clamp protocols and a combination of physiological K^+^‐based external and internal solutions, as described in Section 2. Unfortunately, senescent amniocytes were far more difficult to assess via cytocentering patch‐clamp, presumably because of a stiffer cytoskeleton, therefore we tested only two cells by automated patch‐clamp and 15 cells by manual patch‐clamp with the same solutions and voltage protocols, achieving whole‐cell configuration upon raising bath temperature to 35°C. Because of use of similar whole‐cell approaches, solutions and voltage protocols, the results of automated and manual patch‐clamp experiments are directly comparable. Figure 4 A and B show whole‐cell recordings using the general voltage‐clamp protocol for K^+^ currents performed on two senescent amniocytes via automated patch‐clamp: recording a. shows prominent inactivatable (A‐type) K^+^ current (*I*_A_), while recording b. only delayed outward‐rectifying K^+^ current (*I*_or_). In addition, both recordings show current fluctuations at depolarizing steps suggestive for opening of big conductance Ca^2+^‐dependent K^+^ (BK) channels. In some cryopreserved amniocytes with very small BK currents, individual channel openings are visible, like in a single‐channel recording (Figure 4C). Figure 4D shows voltage‐dependent Na^+^ currents (*I*_Na_), while Figure 4E shows a double voltage ramp recording in a senescent amniocyte, where an inward transient current with activation threshold of approximately −50 mV can be noticed, suggestive of T‐type Ca^2+^ current; this current was recorded in only one cell. Another current component recorded with the double‐ramp protocol was an inward rectifying K^+^ current (*I*_Kir_), visible between −80 and −120 mV, as shown in Figure 5A. The current densities, numbers and percentages of cells where these five current components were identified for cryopreserved and senescent amniocytes are exposed in Table 1. We also studied effects of specific blockers on some of these current components, as shown in Figure 5. Thus, Figure 5A shows the inhibitory effect of iberiotoxin 100 nmol/L on BK channels current fluctuations visible at depolarized potentials in the double‐ramp recording. We quantified this pharmacological effect by computing the square root of current variance *σ* ^2^ (equivalent to standard deviation of the signal—*σ* or SD) in a narrow interval of 6 ms centred on the +80 mV peak potential of the double ramp, where BK channels are maximally activated and the current shows the highest level of fluctuations by channel gating. The average relative SD values of n = 6 experiments measured at 10‐second intervals allowed us to study the kinetics of block by iberiotoxin: via a monoexponential fit we determined a macroscopic apparent time constant *τ *= 13.3 seconds (Figure 5B). We also studied the effect of lidocaine 200 µmol/L on peak Na^+^ current, using a special voltage protocol for use‐dependent block, with a holding potential of −140 mV, to completely remove Na^+^ channels from inactivation, and 40 consecutive 6‐ms depolarizing steps at −20 mV, applied at a frequency of 30 Hz. Figure 5D shows progressive decay in peak *I*_Na_ amplitude produced by lidocaine upon repeated channel opening, and Figure 5C summarizes average relative peak *I*_Na_ values for the first five stimuli of the protocol.

**Table 1:** Features of the main ion current components identified in amniocytes, including TRPM7‐like current explored in separate experiments

Feature	*C*_m_ (pF) (mean ± SD)	*R*_a_ (MΩ) (mean ± SD)	*I*_or_ (pA/pF) (mean ± SD) (at +60 mV)	*I*_A_ (pA/pF) (mean ± SD) (at +60 mV)	BK current fluctuations max. amplitude (pA/pF) (mean ± SD) (at +60 mV)	*I*_Na_ (pA/pF) (mean ± SD) (at −10 mV)	*I*_Kir_ (pA/pF) (mean ± SD) (at −120 mV, base at −80 mV)
Cryopreserved amniocytes (n = 17, automated patch‐clamp) (from n = 6 patients)							
Number of cells			17	9	17	8	9
% of cells			100	53	100	47	53
Mean ± SD	17.8 ± 18.5	12.8 ± 7.1	5.9 ± 4.9	1.4 ± 2.4	5.0 ± 3.0	−1.9 ± 3.3	−3.3 ± 6.2
Senescent amniocytes (n = 17, two via automated and 15 via manual patch‐clamp) (from n = 3 patients)							
Number of cells			17	10	17	7	5
% of cells			100	59	100	41	29
Mean ± SD	52.9 ± 43.8	17.8 ± 13.5	13.8 ± 14.6	5.8 ± 9.1	1.8 ± 1.0	−2.4 ± 4.6	−1.3 ± 4.1
Fisher's exact probability test, *P* value			–	1	‐	1	0.296
Two‐tailed Student's *t* test for independent samples, *P* value			0.04		0.0002		
Two‐tailed Mann‐Whitney test, *P* value	0.0005	0.49		0.31		0.94	0.123

Feature for TRPM7 experiments	*C*_m_ (pF) (mean ± SD)	*R*_a_ (MΩ) (mean ± SD)	Current density at −120 mV (mean ± SD) (difference between DVF and normal cond.)	Current density at +80 mV (mean ± SD) (difference between DVF and normal cond.)	% recovery at −120 mV upon readmission of divalent cations (mean ± SD)	% recovery at +80 mV upon readmission of divalent cations (mean ± SD)
Cryopreserved amniocytes (n = 8, automated patch‐clamp) (from n = 3 patients)						
Mean ± SD	32.4 ± 20.1	9.4 ± 9.7	−1.6 ± 0.8	7.7 ± 7.3	75 ± 38	97 ± 23
Senescent amniocytes (n = 8, manual patch‐clamp) (from n = 3 patients)						
Mean ± SD	23.3 ± 7.0	10.1 ± 4.0	−8.3 ± 7.1	12.8 ± 10.7	100 ± 9	98 ± 14
Two‐tailed Student's *t* test for independent samples, *P* value	0.24		0.0185	0.278	0.095	0.95
Two‐tailed Mann‐Whitney test, *P* value		0.44				

Abbreviations: BK, big conductance Ca^2+^‐dependent K^+^ channels; *C*_m_, membrane capacitance; DVF, divalent‐free; *I*_A_, inactivatable A‐type K^+^ current; *I*_or_, outward rectifier K^+^ current; *I*_Na_, voltage‐dependent Na^+^ current; *I*_Kir_, inward rectifier K^+^ current; *R*_a_, access (series) resistance; TRPM, transient receptor potential melastatin‐related.

### 3.5 Levels of TRPM7 current assessed by patch‐clamp

**Figure 6:**
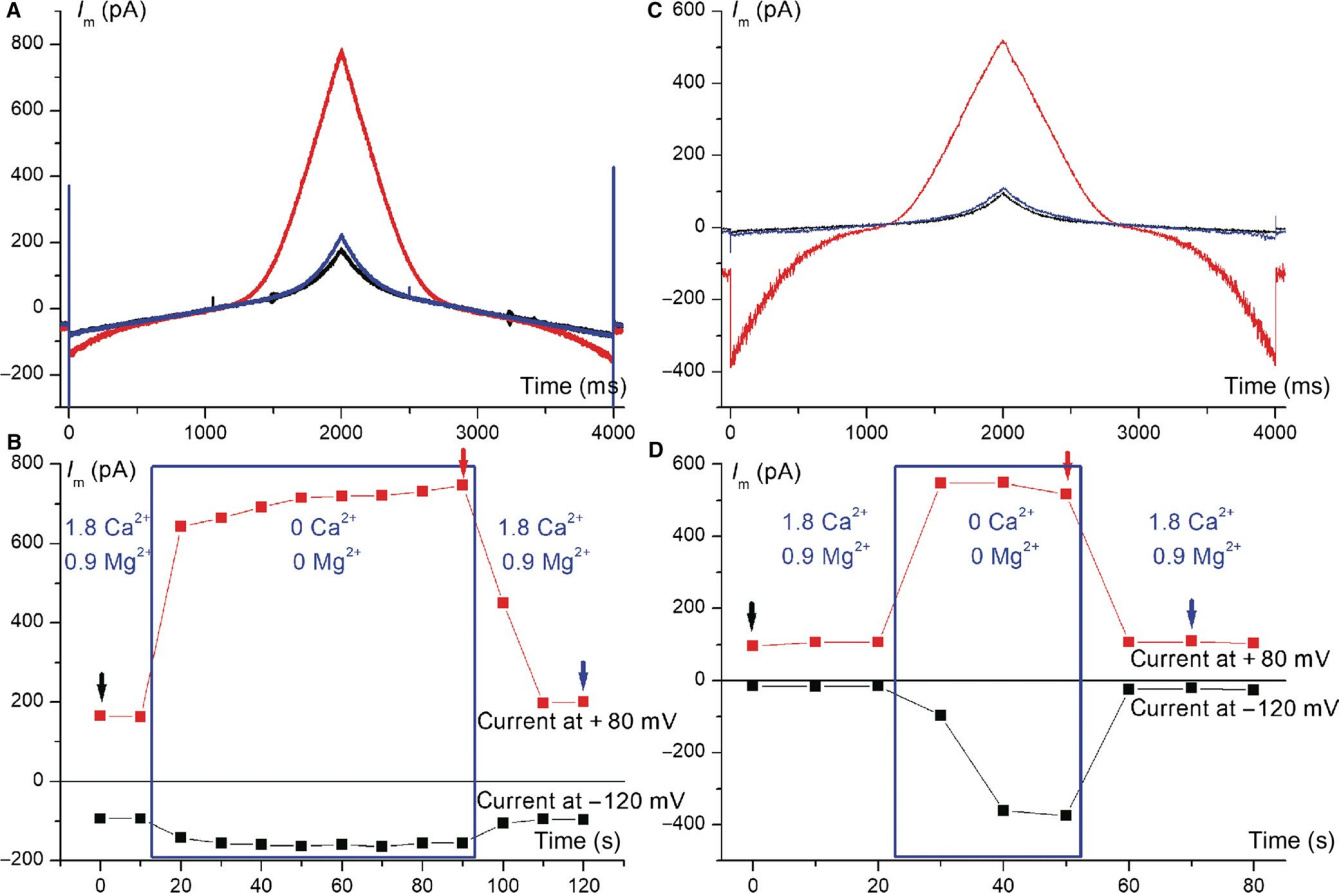
TRPM7‐like currents in amniocytes. (A and C) Current traces recorded with the double‐ramp voltage protocol in a cryopreserved amniocyte (A) and a senescent amniocyte (C) before, during and after divalent cation removal from external solutions (timing is marked with arrows of appropriate colour in B and D, respectively), revealing a large reversible monovalent cation current with a peculiar I‐V plot compatible with TRPM7; (B and D) time course of current amplitudes at −120 and +80 mV during external cation removal in the experiments shown in A and C. TRPM, transient receptor potential melastatin‐related

We studied TRPM7‐like currents in amniocytes using the same double‐ramp voltage protocol and a special combination of Cs^+^‐based external and internal solutions, as shown in Section 2. The internal solution was Mg^2+^‐free and contained ATP, two conditions required for TRPM7 channels activation. As shown in Figure 6A (for a cryopreserved amniocyte) and Figure 6C (for a senescent amniocyte), removal of divalent cations in the external solution unmasked large outward and inward currents carried by monovalent cations, with an I‐V curve similar to that recorded in cardiomyocytes in previous experiments.^49^ Figures 6B and 6D show time plots of current levels at −120 and +80 mV measured on consecutive recordings at 10‐second intervals in these two cells. In both cases perfusion of divalent cation‐free (DVF) external solution produced large currents, and the phenomenon was reversible upon readmission of divalent cations. Table 1 summarizes average TRPM7‐like current densities at −120 and +80 mV (computed as differences between current levels during and before perfusion with divalent‐free solution) and percentages of reversibility for n = 8 cryopreserved and n = 8 senescent amniocytes. Average current densities were higher in senescent amniocytes (for values at −120 mV the difference was statistically significant, *P* = 0.0185, two‐tailed Student's *t* test for independent samples), and the reversibility was generally very good.

## 4 DISCUSSION

The immunophenotype of cryopreserved amniocytes features expression of cellular surface markers CD29, CD44, CD49e, CD54, CD56, CD73, CD90, CD105 and CD146. Interestingly, in senescent amniocytes the expression of these markers is decreased. Putative explanations for this reduced surface marker expression in senescent amniocytes include excessive glycocalix proliferation, senescence‐down‐regulated gene expression, or reduced protein synthesis as an energy‐sparing mechanism, but the real cause remains elusive. The high percentages of cells featuring pluripotency surface antigens SSEA‐1, SSEA‐4, TRA1‐60, TRA1‐81 in both cryopreserved and senescent amniocyte preparations, as well as the presence of pluripotency markers *Oct4* and *Nanog*, prove preferential proliferation of stem cells relative to other cell populations present in amniotic fluid samples during culturing in BIO‐AMF‐2 medium, despite the fact that we have not attempted to isolate or clone stem cells from primary amniotic fluid cellular pools, like in other studies.^51^, ^52^ The differences in percentages of various pluripotency surface antigens can be explained by differences in disappearance kinetics of each marker.^53^

There are at least two major pathways that regulate senescence—the p53/p21 and p16^INK4A^/pRb. Although senescent amniocytes underwent growth arrest, they continued to be metabolically active, with changes in gene expression, morphology, cytoskeleton reorganization, and in activity of SA‐β‐Gal. The molecular pathways triggering senescence involve retinoblastoma protein (pRb) or p53, which activate cyclin‐dependent kinase inhibitors p16 and p21, respectively. Depending on the cellular type, these pathways can be different, but they can also influence each other and cooperate to induce senescence. Other inducers involved in senescence are c‐Myc, p300, Bcl‐2. Moreover, *p16^INK4A^* became a biomarker of aging,^54^ besides its tumour suppressor role. Our results demonstrate high levels of *p16^INK4A^* in senescent cells. Furthermore, MDM2 is a negative regulator of p53 activity, being sequestered by p14arf (the alternate reading frame product of *p16^INK4A^*) in the nucleoli, where it prevents export of p53 from the nucleus to the cytoplasm for degradation by the 26S proteasome subunit.^55^ Senescence is associated with loss of p53 activity that greatly enhances the SASP,^7^ as confirmed by our study; small molecule drugs that can restore function of tumour‐suppressing genes such as *p53* may be used as treatment for senescence‐associated conditions.

Recent data showed that c‐Myc can activate senescence through stromal secretion of TGF‐β.^56^ The SASP is associated with the expression of factors such as IL‐1, IL‐1R, IL‐6, IL‐8, MCPs, MIPs (macrophage inflammatory proteins), TGF‐β. All these molecules initiate a signalling cascade that activates NF‐kB and/or C/EBPβ.^57^ We showed that SASP of human amniocytes includes high levels of *IL‐1*, *IL‐6*, *IL‐8*, *TGF*, *eNOS* and *NF‐κB* (*p65* component). Secretion of these inflammatory factors by senescent cells suggests local or systemic chronic inflammation, which leads to age‐related diseases such as tissue degeneration and hyperplasia. These cytokines also sustain SAGA. Furthermore, the SASP includes at least one alarmin, HMGB1, which can initiate an inflammatory response and interact with p53,^58^ as shown by us via a 2.6‐fold increase in *HMGB1* expression in senescent vs cryopreserved amniocytes.

The electrical phenotype of amniocytes in the present study, obtained via automated/manual whole‐cell patch‐clamp, is similar to that found in our previous studies on human amniocytes.^47^ Using the same voltage protocols and combination of solutions, we found similar percentages of cryopreserved and senescent cells expressing certain ion current components, and similar current densities, with some exceptions. Thus, BK fluctuations were present in all cells included in the present study, and in only ~70% of both cryopreserved and senescent amniocytes in the 2016 study, and the percentage of senescent amniocytes featuring *I*_Kir_ was higher in the present study. The densities of peak *I*_Na_ and *I*_Kir_ in cryopreserved amniocytes were lower when compared to the 2016 study. One possible explanation for this difference could be the use of a different cell culture medium (BIO‐AMF‐2) that better preserves pluripotency and avoids auto/paracrine neural differentiation. The higher levels of TRPM7‐like currents in senescent vs cryopreserved amniocytes may be explained by increased levels of oxidative stress associated with the SASP leading to activation of this current.^59^ The importance of tetraethylammonium‐sensitive delayed outward rectifier K^+^ currents for cell proliferation properties of embryonic and induced pluripotent stem cells has been described.^60^ Similarly, TRPM7 is an ion channel with astounding roles in physiology and physiopathology, including internal Mg^2+^ homeostasis, transmembrane transport of heavy metal ions (Fe^2+^, Zn^2+^, Cu^2+^), regulation of cell proliferation, adhesion and differentiation during embryogenesis,^61^ therefore its increased density in senescent amniocytes may have important functional consequences.

In conclusion, we demonstrated specific changes in senescent vs. cryopreserved amniocytes, including decreased expression of cell surface markers, changes in gene expression associated with SASP and SAGA, positive SA‐β‐Gal staining, and differences in electrical phenotype (ion current densities and percentages of cells featuring a specific ion current component), particularly higher levels of TRPM7‐like inward currents in DVF conditions. Human cultured amniocytes represent an important resource for therapeutic applications because of their high renewal capacity, ability to differentiate along several lineages, combined with low immunogenicity and absence of tumour formation after transplantation.^62^ However, senescence diminishes their anti‐inflammatory properties and renders them pro‐inflammatory, thus limiting their therapeutic potential. In‐depth understanding of senescence mechanisms in MSC is lacking,^39^ but they are of utmost importance in protocols of in vitro cell expansion, which cannot be currently extended beyond a number of cell division cycles and passages. Several treatments have been proposed to prevent in vitro senescence, such as gene editing, Rb silencing, antioxidants (isothiocyanates, 3% hydrogen gas), inhibitors of PI3K(Akt)/mTOR pathways,^40^ offering new perspectives for applications of these valuable stem cells resources.

## CONFLICT OF INTEREST

The authors declare no competing interests related to this study, except for LS being founder and CEO of Genetic Lab S.R.L.

## AUTHOR CONTRIBUTIONS

HM, BA, DA, FI and LS conceived the study. FI and DA performed flow cytometry and qRT‐PCR experiments. RA, BA and FBE performed electrophysiology experiments. LS provided amniocytes. RA, FI, BA, DM and HM interpreted data and prepared the article.
