# Supplementary material for: Senescence‐induced immunophenotype, gene expression and electrophysiology changes in human amniocytes
Source: J Cell Mol Med. 2019 Sep 3;23(11):7233–45. doi: 10.1111/jcmm.14495 (PMC6815807; doi:10.1111/jcmm.14495)
Supplement: Supplementary file 1 [file JCMM-23-7233-s001.DOC]

**Senescence-induced immunophenotype, gene expression and electrophysiology changes in human amniocytes**

Airini R.^1,†^, Iordache F.^2,†^, Alexandru D.^2^, Savu L.^3,4^, Epureanu F.^1^, Mihailescu D.^1^, Amuzescu B.^1,*^, Maniu H.^2^

^1^ Dept. Biophysics & Physiology, Faculty of Biology, University of Bucharest, Splaiul Independentei 91-95, 050095 Bucharest, Romania

^2^ Dept. Regenerative Medicine, “N. Simionescu” Institute of Cell Biology and Pathology, B.P. Hasdeu Str. 8, 050568 Bucharest, Romania

^3^ Genetic Lab S.R.L., Cpt. Av. N. Drossu Str. 9, 012071 Bucharest, Romania

^4^ Fundeni Clinical Institute, Fundeni Rd. 258, 022328 Bucharest, Romania

^†^ equal contribution

^*^ Corresponding author: B. Amuzescu

Dept. Biophysics & Physiology, Faculty of Biology, University of Bucharest

Splaiul Independentei 91-95, Bucharest 050095, Romania

Phone/Fax: +40 21 318 15 73

E-mail: [bogdan@biologie.kappa.ro](mailto:bogdan@biologie.kappa.ro)

**Supporting Information**

**Table S1**. Primer sequences and identification codes for qRT-PCR assays performed in this study.

| **Gene** | **Sequences of oligonucleotide primers** | **Reference** |
| --- | --- | --- |
| *IL-1α* | GCCTCAAGATGAAGGCAAAG (fw)  GATGCCTGGTCACACTCAGA (rv) | Baek *et al*., 2009[1] |
| *IL-6* | CCTCACCCTCCAACAAAGAT (fw)  GCCTCAGACATCTCCAGTCC (rv) | Saban *et al*., 2008[2] |
| *IL-8* | ACTGAGAGTGATTGAGAGTGGAC (fw)  AACCCTCTGCACCCAGTTTTC (rv) | Kostyuk *et al*., 2013[3] |
| *TGF-β* | CCCAGCATCTGCAAAGCTC (fw)  GTCAATGTACAGCTGCCGCA (rv) | Xie *et al*., 2009[4] |
| *HMGB1* | GAGGTGGGAGAGGGAAGGG (fw)  GCAGACATGGTCTTCCACCT (rv) | Own design |
| *NF-κB* (*p65*) | ATCCCATCTTTGACAATCGTGC (fw)  CTGGTCCCGTGAAATACACCTC (rv) | Yi *et al*., 2014[5] |
| *p16^INK4A^* | GACATCCCCGATTGAAAGAA (fw)  TTTACGGTAGTGGGGGAAGG (rv) | Song *et al*., 2008[6] |
| *eNOS* | CCTTCCGCTACCAGCCAGA (fw)  CAGAGATCTTCACTGCATTGGCTA (rv) | Kittel-Schneider *et al*., 2012[7] |
| *β-actin* | AGTGTGACGTTGACATCCGTA (fw)  GCCAGAGCAGTAATCTCCTTCT (rv) | Boteanu *et al*., 2015[8] |
| *Bcl-2* | hs00608023 | ThermoFisher Scientific |
| *c-Myc* | hs00152408 | ThermoFisher Scientific |
| *MDM2* | hs1066930 | ThermoFisher Scientific |
| *p21* | hs00355782 | ThermoFisher Scientific |
| *p53* | hs01034249 | ThermoFisher Scientific |
| *HPRT1* | hs02800695 | ThermoFisher Scientific |
| *Oct4(Pou5f1)* | TGGGGGTTCTATTTGGGAAGG (fw)  GTTCGCTTTCTCTTTCGGGC (rv) | Zhang *et al*., 2010[9] |
| *Nanog* | TGATTCTTCCACCAGTCC (fw)  TGGAGGCTGAGGTATTTC (rv) | Zhang *et al*., 2010[9] |
| *GAPDH* | CCTGCACCACCAACTGCTTA (fw)  GGCCATCCACAGTCTTCTGAG (rv) | He *et al*., 2017[10] |

**Table S2**. Expression levels in qRT-PCR experiments using SYBR Green or TaqMan hydrolysis probes performed on cryopreserved and senescent amniocytes (raw data and average values). Last column indicates *p* values of two-tailed Mann-Whitney test for *C*_T_ raw data for each gene. The *C*_T_ and Δ*C*_T_ columns show the number of replicates for each marker (generally 2 biological replicates i.e. samples form 2 patients, each with 2 technical replicates, for each marker and each condition, but sometimes only one biological replicate per condition or one technical replicate per biological replicate).

| **Gene** | **Cryopreserved amniocytes** | | **Senescent amniocytes** | | **Δ*C*_T_** | | **Fold change** | | ***p*** |
| --- | --- | --- | --- | --- | --- | --- | --- | --- | --- |
|  | *C*_T_ | Mean±SD | *C*_T_ | Mean±SD | Δ*C*_T_ (raw) | Mean±SD | $2^{-\Delta C_{T}}$ | SD |  |
| ***IL-1^α^*** | 31.431 | **32.832**  **±1.454** | 26.871 | **27.387**  **±0.355** | -5.961 | **-5.445**  **±0.355** | **43.562** | **1.279** | **0.029** |
|  | 31.835 |  | 27.587 |  | -5.246 |  |  |  |  |
|  | 33.522 |  | 27.444 |  | -5.389 |  |  |  |  |
|  | 34.540 |  | 27.648 |  | -5.185 |  |  |  |  |
| ***IL-6*** | 33.384 | **33.126**  **±0.227** | 29.028 | **28.726**  **±0.291** | -4.098 | **-4.401**  **±0.291** | **21.119** | **1.223** | **0.029** |
|  | 32.841 |  | 28.733 |  | -4.393 |  |  |  |  |
|  | 33.083 |  | 28.332 |  | -4.794 |  |  |  |  |
|  | 33.197 |  | 28.809 |  | -4.317 |  |  |  |  |
| ***IL-8*** | 28.380 | **28.745**  **±0.560** | 22.373 | **22.344**  **±0.090** | -6.372 | **-6.402**  **±0.090** | **84.544** | **1.064** | **0.029** |
|  | 28.246 |  | 22.321 |  | -6.424 |  |  |  |  |
|  | 29.482 |  | 22.447 |  | -6.298 |  |  |  |  |
|  | 28.873 |  | 22.233 |  | -6.512 |  |  |  |  |
| ***TGF-β*** | 26.183 | **26.378**  **±0.438** | 21.961 | **21.784**  **±0.297** | -4.417 | **-4.594**  **±0.297** | **24.145** | **1.229** | **0.029** |
|  | 25.979 |  | 22.111 |  | -4.266 |  |  |  |  |
|  | 26.993 |  | 21.531 |  | -4.846 |  |  |  |  |
|  | 26.356 |  | 21.534 |  | -4.844 |  |  |  |  |
| ***HMGB1*** | 35.362 | **34.875**  **±0.690** | 32.973 | **33.005**  **±1.835** | -1.902 | **-1.387**  **±1.782** | **2.615** | **3.439** | **0.20** |
|  | 34.085 |  | 31.187 |  | -3.688 |  |  |  |  |
|  | 35.177 |  | 34.856 |  | -0.019 |  |  |  |  |
| ***NF-κB* (*p65*)** | 34.144 | **33.869**  **±0.486** | 33.930 | **31.593**  **±2.147** | -0.061 | **-3.934**  **±3.752** | **15.289** | **13.470** | **0.20** |
|  | 34.155 |  | 29.707 |  | -4.162 |  |  |  |  |
|  | 33.308 |  | 31.142 |  | -2.727 |  |  |  |  |
| ***p16^INK4A^*** | 36.086 | **36.279**  **±0.272** | 27.369 | **27.260**  **±0.154** | -8.910 | **-6.165**  **±3.563** | **71.742** | **11.815** | **-** |
|  | 36.471 |  | 27.151 |  | -9.128 |  |  |  |  |
| ***eNOS*** | 36.307 | **36.503**  **±0.276** | 34.845 | **33.192**  **±2.339** | -1.657 | **-4.464**  **±1.913** | **22.072** | **3.765** | **-** |
|  | 36.698 |  | 31.538 |  | -4.965 |  |  |  |  |
| ***β-actin*** | 20.551 | **20.015**  **±0.443** | 14.700 | **14.537**  **±0.402** | -5.315 | **-5.477**  **±0.402** | **44.551** | **1.322** | **0.029** |
|  | 19.470 |  | 14.095 |  | -5.920 |  |  |  |  |
|  | 20.069 |  | 15.012 |  | -5.003 |  |  |  |  |
|  | 19.968 |  | 14.343 |  | -5.672 |  |  |  |  |
| ***c-Myc*** | 21.189 | **20.921**  **±0.179** | 20.172 | **20.112**  **±0.182** | -0.749 | **-0.809**  **±0.181** | **1.753** | **1.134** | **0.029** |
|  | 20.843 |  | 20.334 |  | -0.587 |  |  |  |  |
|  | 20.815 |  | 19.917 |  | -1.004 |  |  |  |  |
|  | 20.837 |  | 20.023 |  | -0.898 |  |  |  |  |
| ***Bcl-2*** | 25.826 | **25.503**  **±0.564** | 25.188 | **25.029**  **±0.182** | -0.315 | **-0.474**  **±0.182** | **1.389** | **1.135** | **0.34** |
|  | 25.859 |  | 25.179 |  | -0.323 |  |  |  |  |
|  | 25.658 |  | 24.829 |  | -0.673 |  |  |  |  |
|  | 24.667 |  | 24.919 |  | -0.583 |  |  |  |  |
| ***p21*** | 20.067 | **19.918**  **±0.397** | 26.436 | **26.239**  **±0.217** | 6.518 | **6.321**  **±0.217** | **0.013** | **1.162** | **0.029** |
|  | 20.282 |  | 26.372 |  | 6.454 |  |  |  |  |
|  | 19.966 |  | 25.951 |  | 6.033 |  |  |  |  |
|  | 19.357 |  | 26.198 |  | 6.280 |  |  |  |  |
| ***p53*** | 19.652 | **19.795**  **±0.127** | 36.961 | **37.023**  **±0.129** | 17.167 | **17.228**  **±0.130** | **6.5x10^-6^** | **1.094** | **0.029** |
|  | 19.770 |  | 37.190 |  | 17.396 |  |  |  |  |
|  | 19.962 |  | 36.889 |  | 17.095 |  |  |  |  |
|  | 19.794 |  | 37.051 |  | 17.257 |  |  |  |  |
| ***MDM2*** | 20.753 | **20.829**  **±0.086** | 25.998 | **25.902**  **±0.206** | 5.168 | **5.072**  **±0.206** | **0.030** | **1.153** | **0.029** |
|  | 20.880 |  | 26.144 |  | 5.314 |  |  |  |  |
|  | 20.761 |  | 25.702 |  | 4.872 |  |  |  |  |
|  | 20.924 |  | 25.763 |  | 4.934 |  |  |  |  |
| ***HPRT1*** | 24.150 | **24.119**  **±0.067** | 26.072 | **25.968**  **±0.097** | 1.953 | **1.849**  **±0.097** | **0.278** | **1.069** | **0.029** |
|  | 24.031 |  | 25.838 |  | 1.719 |  |  |  |  |
|  | 24.187 |  | 25.989 |  | 1.870 |  |  |  |  |
|  | 24.107 |  | 25.972 |  | 1.853 |  |  |  |  |
| ***OCT4***  ***(Pou5f1)*** | 30.645 | **31.144**  **±0.616** | 28.475 | **28.301**  **±0.134** | 2.669 | **2.844**  **±0.134** | **7.179** | **1.098** | **0.029** |
|  | 31.360 |  | 28.224 |  | 2.920 |  |  |  |  |
|  | 31.919 |  | 28.171 |  | 2.973 |  |  |  |  |
|  | 30.653 |  | 28.332 |  | 2.812 |  |  |  |  |
| ***NANOG*** | 33.390 | **33.274**  **±0.549** | 31.190 | **31.203**  **±0.321** | 2.084 | **2.071**  **±0.321** | **4.202** | **1.249** | **0.029** |
|  | 32.573 |  | 30.843 |  | 2.431 |  |  |  |  |
|  | 33.903 |  | 31.623 |  | 1.651 |  |  |  |  |
|  | 33.228 |  | 31.154 |  | 2.120 |  |  |  |  |
| ***GAPDH*** | 23.240 | **23.301**  **±0.086** | 23.335 | **23.365**  **±0.042** | -0.035 | **-0.064**  **±0.042** | **0.957** | **1.029** | **-** |
|  | 23.361 |  | 23.394 |  | -0.093 |  |  |  |  |

**Table S3**. Percentages of surface markers expression for cryopreserved and senescent amniocytes assessed via flow cytometry (number of replicates in brackets).

| **Marker**  **(fluorophore)** | **% in cryopreserved amniocytes** | **% in senescent amniocytes** | **% difference**  **(cryo.-senescent)** |
| --- | --- | --- | --- |
| CD73 (FITC) | 97.5 (*n* = 3) | 15.2 (*n* = 2) | 82.3 |
| CD90 (PE) | 98.4 (*n* = 3) | 37.7 (*n* = 2) | 60.7 |
| CD105 (FITC) | 67.2 (*n* = 3) | 1.7 (*n* = 2) | 65.5 |
| Integrins | | | |
| CD29 (PE) | 98.4 (*n* = 3) | 54.8 (*n* = 2) | 43.6 |
| CD49e (PE) | 99.8 (*n* = 3) | 34.5 (*n* = 1) | 65.3 |
| Cell adhesion molecules | | | |
| CD31 (PE) | 50.7 (*n* = 3) | 1.5 (*n* = 2) | 49.2 |
| CD44 (FITC) | 98.5 (*n* = 3) | 22.8 (*n* = 2) | 75.7 |
| CD54 (FITC) | 10.3 (*n* = 3) | 8.5 (*n* = 2) | 1.8 |
| CD56 (PE) | 75.2 (*n* = 3) | 12.7 (*n* = 2) | 62.5 |
| CD146 (PE) | 88.7 (*n* = 2) | 58.3 (*n* = 2) | 30.4 |
| Hematopoietic cells markers | | | |
| CD45 (FITC) | 2.9 (*n* = 3) | 3.1 (*n* = 2) | -0.2 |
| CD117 (PE) | 4.9 (*n* = 3) | 1.1 (*n* = 2) | 3.8 |
| CD133 (PE) | 1.7 (*n* = 3) | 2.5 (*n* = 2) | -0.8 |
| Pluripotency cell surface antigens | | | |
| SSEA-1 (PE) | 73.94 (*n* = 2) | 58.3 (*n* = 2) | 15.64 |
| SSEA4-(FITC) | 84.05 (*n* = 2) | 71.5 (*n* = 2) | 12.55 |
| TRA1-60 (PE) | 92.9 (*n* = 2) | 91.35 (*n* = 2) | 1.55 |
| TRA1-81 (PE) | 90 (*n* = 2) | 84.55 (*n* = 2) | 5.45 |

FITC - fluorescein isothiocyanate

PE - phycoerythrin

**Figure S1.** Supplementary cell surface markers assessed via flow cytometry in cryopreserved and senescent amniocytes, as well as negative isotype sera IgG FITC and IgG PE, used to define the gates. Percentages of positive cells for each marker are indicated on the corresponding distribution histogram.

**
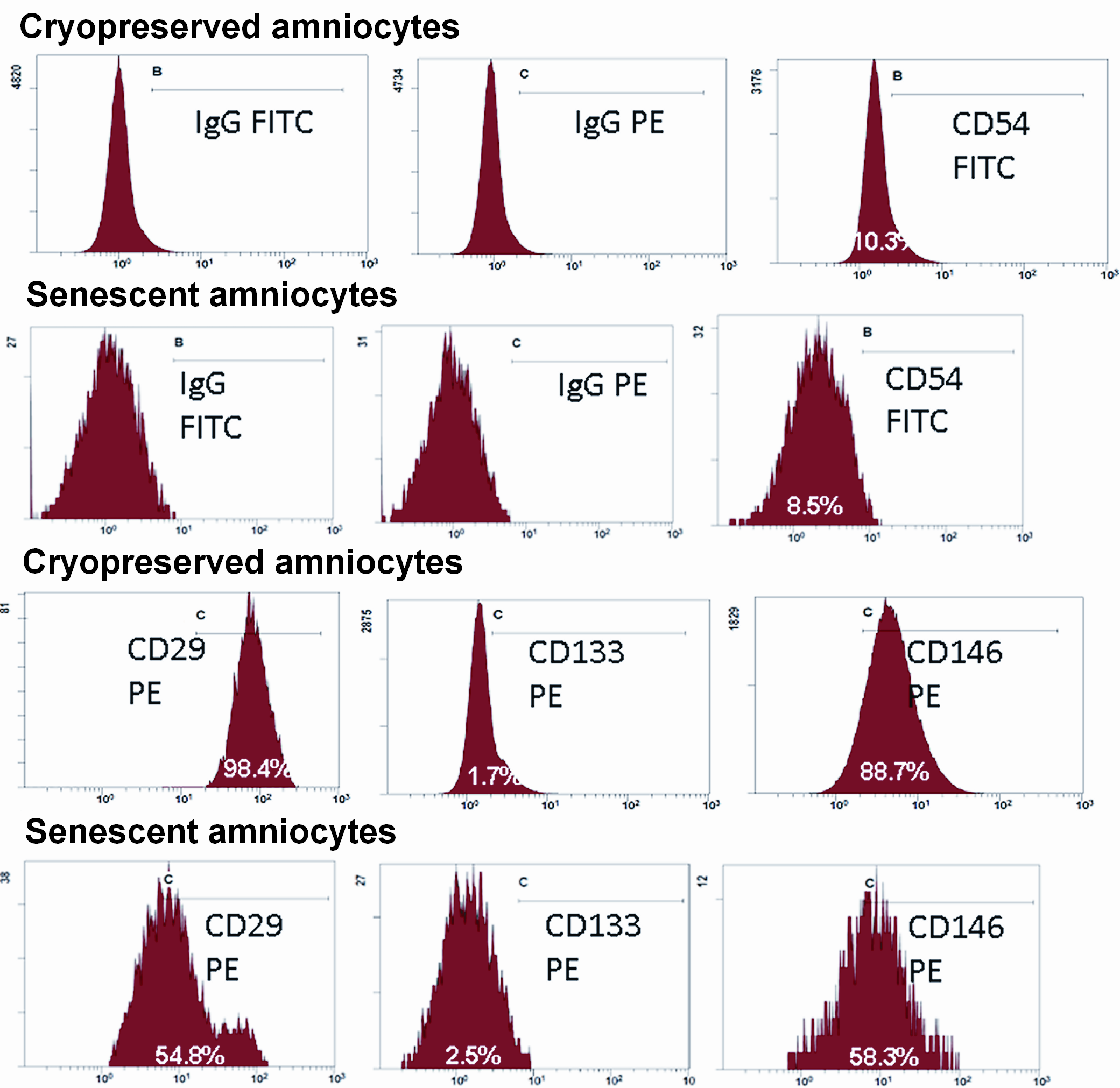
**

**Figure S2.** Supplementary pluripotency cell surface markers assessed via flow cytometry in cryopreserved and senescent amniocytes. Percentages of positive cells for each marker are indicated on the corresponding distribution histogram.


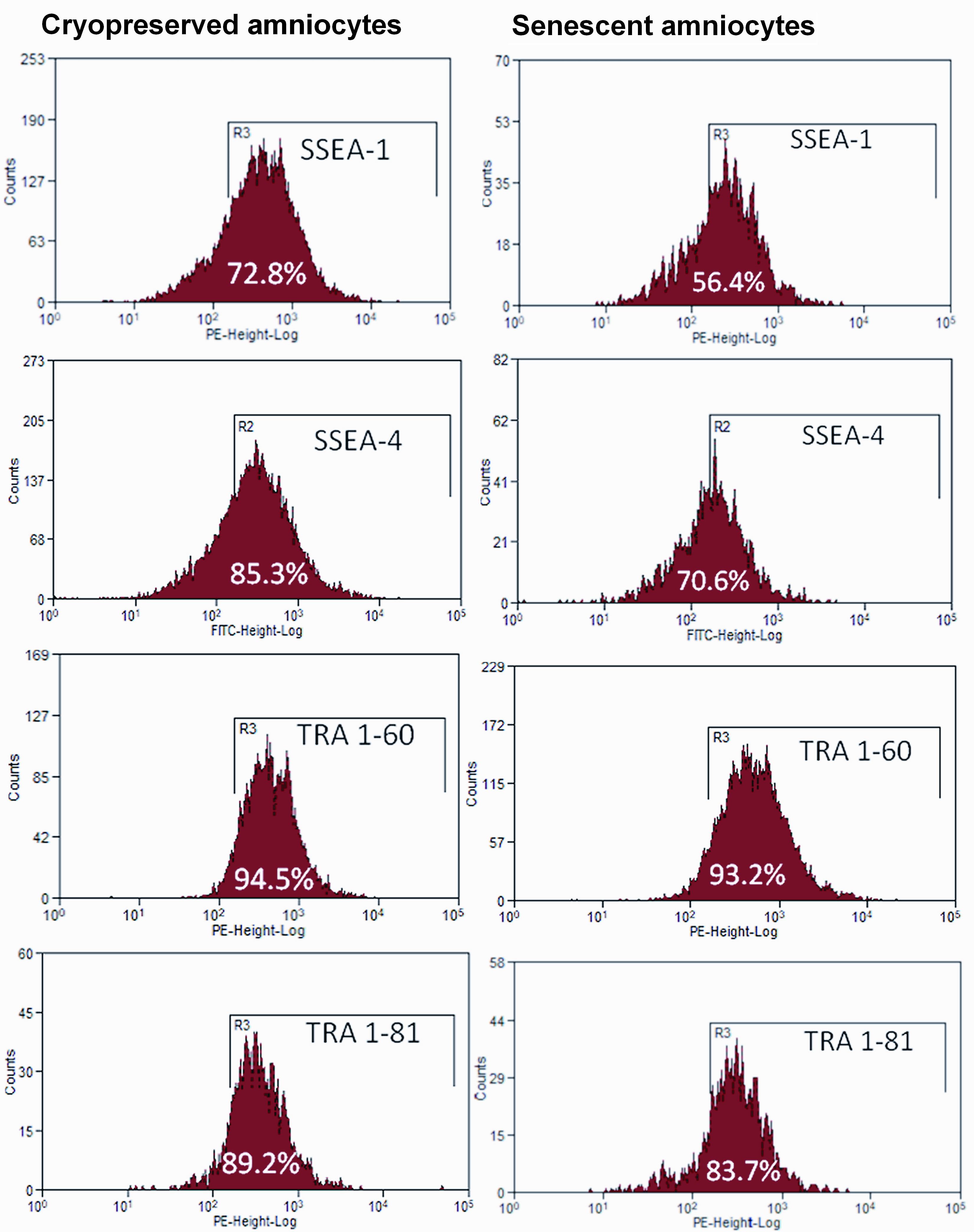


**Supplementary References**

1. **Baek YS, Haas S, Hackstein H, Bein G, Hernandez-Santana M, Lehrach H, Sauer S, Seitz H.** Identification of novel transcriptional regulators involved in macrophage differentiation and activation in U937 cells. *BMC Immunol*. 2009; 10:18.: 10.1186/471-2172-10-18.

2. **Saban MR, O'Donnell MA, Hurst RE, Wu XR, Simpson C, Dozmorov I, Davis C, Saban R.** Molecular networks discriminating mouse bladder responses to intravesical bacillus Calmette-Guerin (BCG), LPS, and TNF-alpha. *BMC Immunol*. 2008; 9:4.: 10.1186/471-2172-9-4.

3. **Kostyuk SV, Konkova MS, Ershova ES, Alekseeva AJ, Smirnova TD, Stukalov SV, Kozhina EA, Shilova NV, Zolotukhina TV, Markova ZG, Izhevskaya VL, Baranova A, Veiko NN.** An exposure to the oxidized DNA enhances both instability of genome and survival in cancer cells. *PLoS One*. 2013; 8: e77469.

4. **Xie S, Macedo P, Hew M, Nassenstein C, Lee KY, Chung KF.** Expression of transforming growth factor-beta (TGF-beta) in chronic idiopathic cough. *Respir Res*. 2009; 10:40.: 10.1186/465-9921-10-40.

5. **Yi B, Hu X, Zhang H, Huang J, Liu J, Hu J, Li W, Huang L.** Nuclear NF-kappaB p65 in peripheral blood mononuclear cells correlates with urinary MCP-1, RANTES and the severity of type 2 diabetic nephropathy. *PLoS One*. 2014; 9: e99633.

6. **Song Z, Wang Y, Xie L, Zang X, Yin H.** Expression of senescence-related genes in human corneal endothelial cells. *Mol Vis*. 2008; 14: 161-70.

7. **Kittel-Schneider S, Kenis G, Schek J, van den Hove D, Prickaerts J, Lesch KP, Steinbusch H, Reif A.** Expression of monoamine transporters, nitric oxide synthase 3, and neurotrophin genes in antidepressant-stimulated astrocytes. *Front Psychiatry*. 2012; 3:33.: 10.3389/fpsyt.2012.00033. eCollection 2012.

8. **Boteanu RM, Uyy E, Suica VI, Antohe F.** High-mobility group box 1 enhances the inflammatory process in diabetic lung. *Arch Biochem Biophys*. 2015; 583: 55-64.

9. **Zhang S, Geng H, Xie H, Wu Q, Ma X, Zhou J, Chen F.** The heterogeneity of cell subtypes from a primary culture of human amniotic fluid. *Cell*. 2010; 15: 424-39.

10. **He X, Jiang W, Luo Z, Qu T, Wang Z, Liu N, Zhang Y, Cooper PR, He W.** IFN-gamma regulates human dental pulp stem cells behavior via NF-kappaB and MAPK signaling. *Sci Rep*. 2017; 7.
